# EHMTI-0181. Fast recovery of visual evoked potential amplitude after photostress in migraine patients between attacks

**DOI:** 10.1186/1129-2377-15-S1-E9

**Published:** 2014-09-18

**Authors:** G Coppola, D Di Lenola, M Bracaglia, G Di Ciaccia, C Di Lorenzo, V Parisi, F Pierelli

**Affiliations:** 1 Department of Neurophysiology of Vision and Neurophthalmology, G.B. Bietti Foundation-IRCCS, Roma, Italy; 2 Department of medico-surgical sciences and biotechnologies, Sapienza University of Rome Polo Pontino, Latina, Italy

## Background

Subtle impaired macular vision was observed among different psychophysical experimental tasks in migraine patients.

## Aim

Here we studied visual evoked potential (VEP) after photostress (PS) representing an objective index of the dynamic properties of macular performance after exposure to intense light stimulation.

## Method

We recorded VEPs in basal condition and after PS in 43 migraine patients (19 with [MA] and 24 without [MO] aura) and 14 healthy volunteers (HV). PS is induced for a duration of 30s by means of the bulb of a 200-W lamp. We compared P100 implicit time and N75-P100 amplitude of baseline VEP with those collected every 20s up to 200s after PS.

## Results

VEP parameters did not differed between groups at the baseline. In all groups, the VEPs recorded after PS showed a significant increase in latency at 20s. In HV, N75-P100 amplitude significantly decreased 20s after PS, and recovered subsequently. There was no effect in the migraine groups. In fact, the percentage reduction in N75-P100 amplitude observed at 20s after photostress in MO and MA patients were lower than in HV (MO & MA vs. HV P<0.05). When data of migraine patients were combined, the percentage of amplitude change at 20s was negatively correlated with number of days since the last migraine attack (r=-0.525, p=0.02).

## Conclusion

We documented altered recovery after PS under the influence of imminent attack. Whether or not present VEP findings are related to the ictal/interictal migraineur susceptibility to abnormal sensory perception, such as visual discomfort, remains to be determined.

No conflict of interest.

